# Supplementary material for: Evidence for an atomic chiral superfluid with topological excitations
Source: Nature. 2021 Aug 11;596(7871):227–31. doi: 10.1038/s41586-021-03702-0 (PMC8357630; doi:10.1038/s41586-021-03702-0)
Supplement: Supplementary file 1 — This file contains supplementary text, supplementary equations s1 – s31, supplementary figures s1 – s7 and supplementary references. [file 41586_2021_3702_MOESM1_ESM.pdf]

nature portfolio

https://doi.org/10.1038/s41586-021-03702-0

Supplementary information

Evidence for an atomic chiral superfluid with topological excitations

In the format provided by the authors and unedited

Supplementary Information for ”Evidence for an atomic chiral superfluid with topological excitations”

Xiao-Qiong Wang, Guang-Quan Luo, Jin-Yu Liu, W. Vincent Liu,
Andreas Hemmerich, Zhi-Fang Xu

S-1 Experimental realization of Boron-Nitride optical lattice

A two-dimensional (2D) hexagonal Boron-Nitride (BN) optical lattice potential is created by three laser beams, propagating in the xy-plane and intersecting at $120°$ angles. Each laser beam comprises two frequency components ω_1 and ω_2, both linearly polarized along the z-direction, with wavelength $\lambda \simeq 1064$ nm, and hence with negative detuning with respect to the relevant atomic transitions of rubidium atoms at 780 nm and 795 nm. The two frequency components are derived from two independent lasers. Experimentally, we first combine the two laser beams at frequencies ω_1 and ω_2, respectively, before splitting them into three beams. The total electric field for all beams is then written as

$$\mathbf{E}(\mathbf{r},t) = E_1\mathbf{e}_z \sum_j \cos\left(\mathbf{k}_j \cdot \mathbf{r} - \omega_1 t + \theta_j\right) + E_2\mathbf{e}_z \sum_j \cos\left(\mathbf{k}'_j \cdot \mathbf{r} - \omega_2 t + \theta'_j\right). \tag{S1}$$

Here, the wave vectors are given by $\mathbf{k}_1 = k_L(-\sqrt{3}/2, 1/2)$, $\mathbf{k}_2 = k_L(\sqrt{3}/2, 1/2)$, $\mathbf{k}_3 = k_L\,(0,-1)$, and $\mathbf{k}'_j = (\omega_2/\omega_1)\mathbf{k}_j$, where $k_L = \omega_1/c$. $\theta_j = \omega_1 L_j/c + \theta_0$ and $\theta'_j = \omega_2 L_j/c + \theta'_0$, where L_j denotes the optical path length of the j-th beam from the splitting point to the center of the lattice. The corresponding laser intensity $I(\mathbf{r})$ is proportional to the time averaging of the

square of the electric field according to

$$
\begin{aligned}
I(\mathbf{r}) \propto & \frac{3}{2} E_1^2 + E_1^2 \sum_{\langle i,j \rangle} \cos\left[(\mathbf{k}_i - \mathbf{k}_j) \cdot \mathbf{r} + \theta_i - \theta_j\right] \\
& + \frac{3}{2} E_2^2 + E_2^2 \sum_{\langle i,j \rangle} \cos\left[\left(\mathbf{k}'_i - \mathbf{k}'_j\right) \cdot \mathbf{r} + \theta'_i - \theta'_j\right],
\end{aligned} \tag{S2}
$$

where the summation $\langle i, j\rangle$ is limited to $\langle 1, 2\rangle$, $\langle 2, 3\rangle$, $\langle 3, 1\rangle$. The generated optical lattice potential is proportional to the laser intensity and takes the form

$$
\begin{aligned}
V_{\mathrm{BN}}(\mathbf{r}) = & - V_1 \left\{ 3 + 2 \sum_{\langle i,j \rangle} \cos\left[(\mathbf{k}_i - \mathbf{k}_j) \cdot \mathbf{r} + (\theta_i - \theta_j)\right] \right\} \\
& - V_2 \left\{ 3 + 2 \sum_{\langle i,j \rangle} \cos\left[\left(\mathbf{k}'_i - \mathbf{k}'_j\right) \cdot \mathbf{r} + \left(\theta'_i - \theta'_j\right)\right] \right\},
\end{aligned} \tag{S3}
$$

where $V_{1,2} \geq 0$ for the relevant case of red detuning. Each of the two spectral components creates a triangular lattice potential, which sum up to form the total potential V_{BN}. No interference terms arise since the frequency difference $\Delta\omega = \omega_1 - \omega_2$ is chosen in the range of a few GHz, which exceeds by far all relevant time-scales of the atom dynamics. The relative position of the two triangular lattices is determined by the phase differences

$$
\Delta\theta_{ij} = (\theta_i - \theta_j) - \left(\theta'_i - \theta'_j\right) = \frac{\omega_1 - \omega_2}{c} (L_i - L_j). \tag{S4}
$$

Note that with $\Delta\omega \approx 2\pi \times 3\,\mathrm{GHz}$, a change of the lengths L_i on the order of $10\,\mu m$ corresponds to irrelevant changes of $\Delta\theta_{ij}$ on the order of $10^{-4} \times 2\pi$. Hence, $\Delta\theta_{ij}$ can be readily adjusted without the need of interferometric control of the lengths L_i. Experimentally, we choose convenient values for the lengths L_1, L_2 and L_3 and lock the frequency difference between the two lasers accordingly to fine-tune the relative position of the two triangular lattices appropriately to generate the desired BN optical lattice. We set $(L_1 - L_2, L_2 - L_3, L_3 - L_1) = (-6.04, 3.02, 3.02)$ cm and $\Delta\omega = \omega_1 - \omega_2 = 2\pi \times 3.308$ GHz, which leads to

$(\Delta\theta_{12}, \Delta\theta_{23}, \Delta\theta_{31}) = (-4\pi/3, 2\pi/3, 2\pi/3)$ and hence the BN lattice potential

$$\begin{aligned} V_{\mathrm{BN}}(\mathbf{r}) = &- V_1 \left\{ 3 + 2 \sum_{\langle i,j \rangle} \cos\left[(\mathbf{k}_i - \mathbf{k}_j) \cdot \mathbf{r} - \frac{2\pi}{3} \right] \right\} \\ &- V_2 \left\{ 3 + 2 \sum_{\langle i,j \rangle} \cos\left[(\mathbf{k}_i - \mathbf{k}_j) \cdot \mathbf{r} + \frac{2\pi}{3} \right] \right\}. \end{aligned} \tag{S5}$$

The first (second) term describes the triangular lattice potential that gives rise to the $\mathcal{A}$-sites ($\mathcal{B}$-sites) in the combined BN lattice shown in Fig. 1a of the main text. The potential difference between $\mathcal{A}$-wells and $\mathcal{B}$-wells can be readily adjusted on the μs time-scale via tuning the ratio V_1/V_2. To avoid deformations of the lattice potential, the relative frequency difference between the two sets of triangular lattices and the laser intensities are carefully stabilized.

S-2 Loading of lattice, time and energy scales, and detection schemes

A BEC of typical 4×10^4 ^{87}Rb atoms in the state $|F = 1, m_F = -1\rangle$ is prepared in an optical dipole trap formed by two crossed laser beams with trapping frequencies of $\{\omega_x, \omega_y, \omega_z\} = 2\pi \times \{26.4(1), 26.7(1), 70.6(3)\}$ Hz. Including the gravitational force, pointing into the $-z$-direction, the trap depth along the $-z$-direction is $34\,$nK. A bias magnetic field of 1 G is applied along the z-axis. Within $120\,$ms, the lattice beam intensity is ramped up to $V_1 = 7.04\ E_{\mathrm{R}}$ and $V_2 = 8.03\ E_{\mathrm{R}}$, where $E_{\mathrm{R}} = h^2/2m\lambda^2$. At this stage the overall trap depth along the $-z$-direction is $221\,$nK. A crucial step in our atom preparation protocol is additional evaporative cooling of the atoms after they are loaded to the lattice potential. Therefore, after 5 ms, the depth of the optical dipole trap is ramped down in $15\,$ms, such that the overall trap depth along the $-z$-direction is reduced to $41\,$nK. This enables a forced escape of energetic atoms and thermalization of remaining atoms. Excitation into the second band is obtained by swapping the depths of the $\mathcal{A}$ and $\mathcal{B}$ sites via linearly changing (V_1, V_2) to $(7.81, 7.23)\ E_{\mathrm{R}}$ rapidly in

$0.1\,\mathrm{ms}$. This time is short as compared to the tunneling time ($\approx 1.7\,\mathrm{ms}$) and long compared to the onsite oscillation time scale of about 10μs, determined by the gap between the first and second bands. The trap depth along the $-z$-direction is thereby further reduced to $24\,\mathrm{nK}$, which gives rise to further evaporation.

We record momentum spectra via time-of-flight (TOF) spectroscopy or apply a band mapping technique that allows us to observe the quasi-momentum distribution. These techniques are used to derive the data in Fig. 2c of the main text. To derive the data in Fig. 4c of the main text, the experimental protocol is slightly extended. After excitation to the second band and a subsequent holding time of $205\,\mathrm{ms}$, the p-orbitals in the $\mathcal{A}$-wells are lowered by continuously increasing V_1 to $8.35\,E_\mathrm{R}$ in 1 ms. Therefore, atoms are transferred from the s-orbitals in the shallow $\mathcal{B}$ site to nearby p-orbitals. Momentum spectra of the atoms in the xy-plane are obtained by switching off all potentials in less than $1\,\mu$s and subsequently allowing for a $20\,\mathrm{ms}$ long ballistic expansion before performing absorption imaging. For band mapping measurements, we decrease the intensity of the lattice exponentially with a time constant of $260\,\mu$s followed by $20\,\mathrm{ms}$ of ballistic expansion before performing absorption imaging. For the atom loss data shown in Fig. 3c of the main text, we perform in-situ absorption imaging of a plane perpendicular to the xy-plane, after the atoms are excited to the second band and held there during a variable time.

Here we provide some relevant time and energy scales. After evaporation cooling, the peak density of the BEC in the dipole trap (with trap depth $34\,\mathrm{nK}$ in z-direction including gravity) is about $n_0 \approx 4.7 \times 10^{13}\,\mathrm{cm}^{-3}$. The corresponding mean-field interaction energy is $E_\mathrm{col} = n_0\, 4\pi\hbar^2\, a_s/m$ with the s-wave scattering length $a_s \approx 100.4\, a_0$ ($a_0 =$ Bohr radius, $m =$ rubidium mass), i.e., $E_\mathrm{col}/\hbar = 2\pi \times 365\,\mathrm{Hz}$. We estimate the temperature as roughly about $1/4$ of the trap depth of $34\,\mathrm{nK}$, i.e. $T \approx 10\,\mathrm{nK}$. The two-body collision parameter is $\beta \equiv \sigma\, \bar{v}$ with $\sigma = 8\pi a_s^2$ and $\bar{v} = \sqrt{16 k_B T/(\pi\, m)}$. The associated collision time is $\tau = 1/(\beta n_0) = 14\,\mathrm{ms}$,

which is compatible with the observed time for coherence build-up of a few ten ms. For $(V_1, V_2) = (7.81, 7.23)\, E_\mathrm{R}$, the tight-binding tunneling amplitude between adjacent s and p orbitals is $0.289 E_\mathrm{R}$ (cf. Figure S3), which corresponds to a tunneling time of about $1.7\,\mathrm{ms}$.

S-3 Band structure and Bose-Hubbard model

The BN lattice is spanned by the primitive vectors $\mathbf{a}_1 = a(\sqrt{3}/2, 1/2)$ and $\mathbf{a}_2 = a(0,1)$ with $a = 4\pi/(3k_L)$ denoting the lattice constant, which gives rise to a hexagonal unit cell with alternating potential minima at the corners, denoted $\mathcal{A}$ and $\mathcal{B}$ in Fig. 1a of the main text. Experimentally, the lattice intensity parameters $V_{1,2}$ can be tuned to independently adjust the lattice depths and potential offsets of the $\mathcal{A}$- and $\mathcal{B}$-wells. When $V_1 = V_2$, the lattice potential exhibits D_6 point group symmetry, while in general case with $V_1 \neq V_2$, the point group symmetry is reduced to D_3. The corresponding reciprocal lattice vectors are $\mathbf{b}_1 = \sqrt{3}k_L(1,0)$ and $\mathbf{b}_2 = \sqrt{3}k_L(-1/2, \sqrt{3}/2)$. The associated hexagonal first Brillouin zone (BZ) comprises different points of high symmetry: the Γ point located at its origin, two inequivalent K points labeled as $K_\vartriangle$ and $K_\triangledown$, at the corners and three inequivalent M points centered between adjacent K points (c.f. Fig. 1b of main text).

We apply a plane-wave expansion to numerically determine the band structure. We use more than 200 plane waves, which is sufficient for the low-energy bands. Figure S1 shows the energy spectra for two cases: (1) $V_1 = 7.81\, E_\mathrm{R}$ and $V_2 = 7.23\, E_\mathrm{R}$; (2) $V_1 = 8.35\, E_\mathrm{R}$ and $V_2 = 7.23\, E_\mathrm{R}$. As the 2nd, 3rd, and 4th bands are isolated from other bands, we consider only these three bands to describe the atomic dynamics after loading atoms into the second band via the band swapping technique. The corresponding orbitals are s-orbitals in the shallow $\mathcal{B}$ sites and two p-orbitals in the deeper $\mathcal{A}$ sites. For both cases, we notice that a quadratic band crossing point appears at the Γ point. This degeneracy is protected by the D_3 point-group symmetry of the lattice and the time-reversal symmetry of the system. As we discuss below, when atoms condense in

the second band at one of the K points, interactions induce the spontaneous breaking of time-reversal symmetry, which leads to a topological gap opening at the associated quadratic band crossing point in the Bogoliubov excitation bands of the condensate.

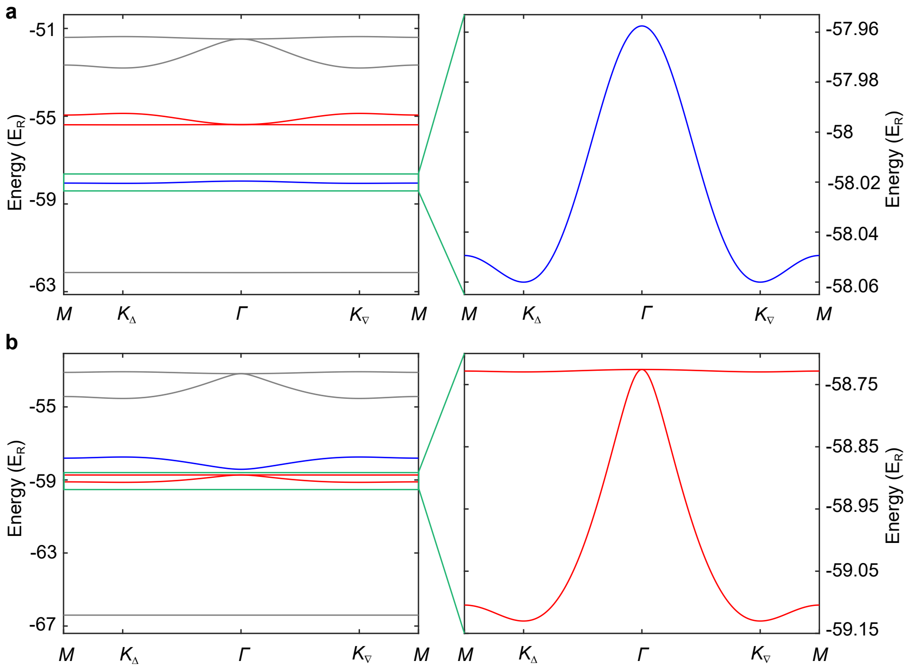

Figure S1: The single-particle energy bands with (**a**) $V_1 = 7.81E_{\rm R}$ and $V_2 = 7.23E_{\rm R}$, and (**b**) $V_1 = 8.35E_{\rm R}$ and $V_2 = 7.23E_{\rm R}$. The blue band is mainly contributed from s orbitals in the shallow lattice sites and the two red bands are mainly contributed from p orbitals in deep lattice sites.

Our system can also be described by a tight-binding model, using a basis of localized Wannier functions. Such Wannier functions are not uniquely defined, however, the most suitable choice are the maximally-localized Wannier functions, which exhibit the least spatial spread, such that higher order tunneling amplitudes are minimized. We obtain such Wannier functions via the Marzari-Vanderbilt method numerically [1]. Direct numerical calculation considering the bands 2,3,4 gives rise to three real Wannier functions $w_s(\mathbf{r})$, $w_{p_x}(\mathbf{r})$ and $w_{p_y}(\mathbf{r})$, which represent s-orbitals located at shallow lattice sites and p_x and p_y orbitals located at deep lattice sites, as shown in Fig. S2. We note that the shapes of the Wannier functions are very different

from the general spherical harmonic functions due to the anisotropic lattice potential, especially for the p orbitals. The Wannier function for the s orbitals exhibit three-fold rotational symmetry (c.f. Fig. S2a), and the Wannier functions for the p_x and p_y orbitals maintain the up-down symmetry but lose the left-right symmetry (c.f. Fig. S2b,c).

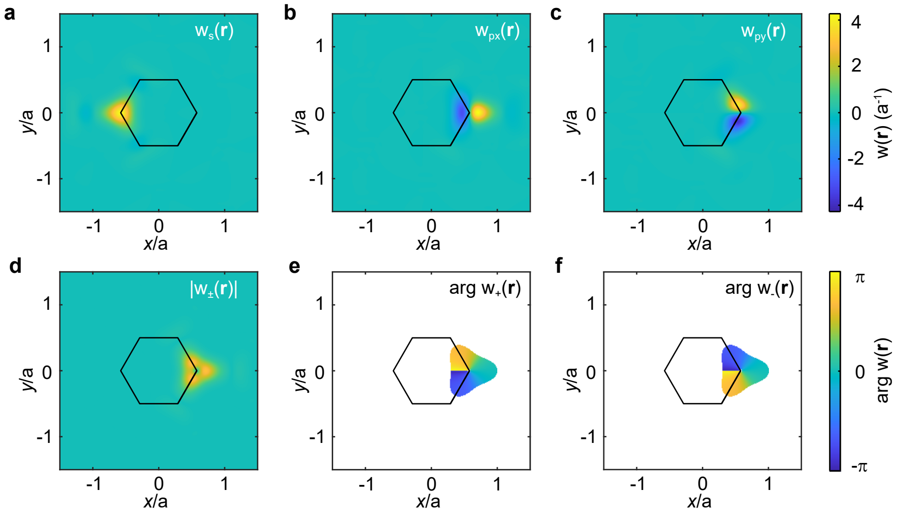

Figure S2: (**a**, **b**, **c**) Real Wannier functions for (**a**) s orbital, (**b**) p_x orbital and (**c**) p_y orbital. (**d**) Modulus of Wannier functions $w_{\pm}(\mathbf{r})$. (**e**, **f**) Phase distribution for Wannier functions (**e**) $w_+(\mathbf{r})$ and (**f**) $w_-(\mathbf{r})$. The black solid honeycombs indicate the unit cell.

The unusual, asymmetrical p-orbital Wannier functions impede an easy implementation of a tight-binding model in the traditional bases of s, p_x and p_y orbitals. To overcome this problem, we introduce a pair of complex Wannier functions $w_{\pm}(\mathbf{r}) = [w_{p_x}(\mathbf{r}) \pm i w_{p_y}(\mathbf{r})]/\sqrt{2}$, which satisfy the relationship $w_+(\mathbf{r}) = w_-^*(\mathbf{r})$ and possess three-fold rotational symmetry. The complex Wannier functions exhibit counter-clockwise and clockwise phase rotations, corresponding to orbital angular momenta $+\hbar$ and $-\hbar$, respectively. Using the Wannier basis $w_s(\mathbf{r})$, $w_+(\mathbf{r})$ and $w_-(\mathbf{r})$, we thus obtain a Bose-Hubbard model to describe the atoms moving in the higher bands of the BN lattice. For the single-particle part, we consider a tight-binding model involving only nearest-neighbor tunnelings, as shown in Fig. S3 (**a**). The single-particle Hamiltonian in real

space is given by

$$\hat{H}_0 = \sum_{\mathbf{r}\in B} \varepsilon_s \hat{s}^\dagger_{\mathbf{r}} \hat{s}_{\mathbf{r}} + \sum_{\mathbf{r}\in A} \varepsilon_p \left(\hat{p}^\dagger_{+,\mathbf{r}} \hat{p}_{+,\mathbf{r}} + \hat{p}^\dagger_{-,\mathbf{r}} \hat{p}_{-,\mathbf{r}}\right) + \sum_{\mathbf{r}\in B,j} \left(t_j \hat{s}^\dagger_{\mathbf{r}} \hat{p}_{+,\mathbf{r}+\mathbf{e}_j} + t_j^* \hat{s}^\dagger_{\mathbf{r}} \hat{p}_{-,\mathbf{r}+\mathbf{e}_j} + \text{h.c.}\right). \tag{S6}$$

Here, $\hat{s}$ and $\hat{p}_\pm$ are annihilation operators for s and $p_\pm$ orbitals, respectively. $t_1 = -t$, $t_2 = -te^{i2\pi/3}$, $t_3 = -te^{-i2\pi/3}$ and $\mathbf{e}_1 = a_0(-1, 0)$, $\mathbf{e}_2 = a_0\left(1/2, -\sqrt{3}/2\right)$, $\mathbf{e}_3 = a_0\left(1/2, \sqrt{3}/2\right)$, $j = 1, 2, 3$ and $a_0 = 4\pi/(3\sqrt{3}k_L)$ is the distance between two nearest-neighbor lattice sites. After performing a Fourier transformation, the single-particle Hamiltonian in momentum space can be written as $\hat{H}_0 = \sum_{\mathbf{k}} \hat{\Psi}^\dagger_{\mathbf{k}} \mathcal{H}_0(\mathbf{k}) \hat{\Psi}_{\mathbf{k}}$, where $\hat{\Psi}_{\mathbf{k}} = (\hat{s}_{\mathbf{k}},\ \hat{p}_{+,\mathbf{k}},\ \hat{p}_{-,\mathbf{k}})^{\mathrm{T}}$ and

$$\mathcal{H}_0(\mathbf{k}) = \begin{pmatrix} \varepsilon_s & \sum_j t_j e^{i\mathbf{k}\cdot\mathbf{e}_j} & \sum_j t_j^* e^{i\mathbf{k}\cdot\mathbf{e}_j} \\ & \varepsilon_p & 0 \\ \text{h.c.} & & \varepsilon_p \end{pmatrix}. \tag{S7}$$

The parameters in the tight-binding model are numerically calculated with the help of the Wannier functions. Figure S3 (**B**) and (**C**) show the corresponding energy spectra obtained by diagonalizing $\mathcal{H}_0(\mathbf{k})$ of Eq. (S7) for the two cases illustrated in Fig. S1. For the first case, the s-orbitals in the shallow wells have lower energy than the p-orbitals in the deep wells with $\varepsilon_s < \varepsilon_p$. The second band of the BN lattice (lowest band of the tight-binding model) has more contribution from the s-orbitals and the degeneracy occurs between the 3rd and the 4th bands. For the second case, the p-orbitals are lower in energy than the s orbitals with $\varepsilon_p < \varepsilon_s$. Thus, the second band of the BN lattice has more contribution from the p-orbitals and the degeneracy occurs between the 2nd and the 3rd bands. Nevertheless, our results coincide with the exact band structure calculated via plane wave expansion. Including more tunneling coefficients, such as adding next and next next nearest neighbor hopping, the energy spectra perfectly match with the exact values.

Considering the contact s-wave interaction among ^{87}Rb atoms, we can write the Hamilto-

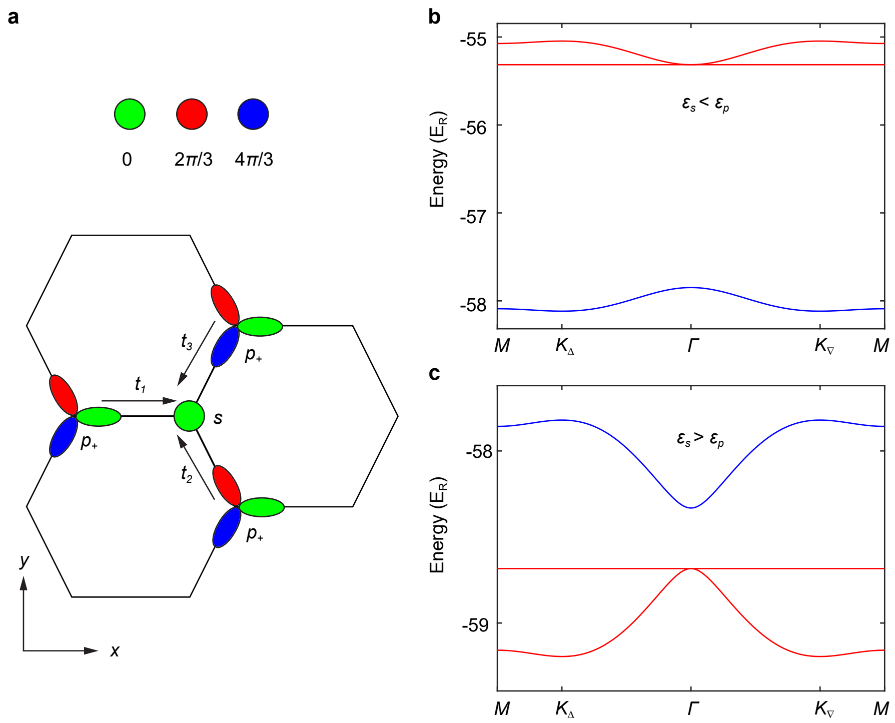

Figure S3: (**a**) Schematic illustration of the tight-binding model. (**b**, **c**) The energy spectra for two different cases: (**b**) $\varepsilon_s = -57.8488E_\mathrm{R}$, $\varepsilon_p = -55.3149E_\mathrm{R}$, and $t = 0.2891E_\mathrm{R}$ and (**c**) $\varepsilon_s = -58.3315E_\mathrm{R}$, $\varepsilon_p = -58.6837E_\mathrm{R}$, and $t = 0.2213E_\mathrm{R}$.

nian of the interaction part as

$$\hat{H}_{\text{int}} = \sum_{\mathbf{r}\in B} \frac{U_s}{2} \hat{s}^\dagger_{\mathbf{r}} \hat{s}^\dagger_{\mathbf{r}} \hat{s}_{\mathbf{r}} \hat{s}_{\mathbf{r}} + \frac{U_p}{2} \sum_{\mathbf{r}\in A} \left(\hat{p}^\dagger_{+,\mathbf{r}} \hat{p}^\dagger_{+,\mathbf{r}} \hat{p}_{+,\mathbf{r}} \hat{p}_{+,\mathbf{r}} + \hat{p}^\dagger_{-,\mathbf{r}} \hat{p}^\dagger_{-,\mathbf{r}} \hat{p}_{-,\mathbf{r}} \hat{p}_{-,\mathbf{r}} + 4 \hat{p}^\dagger_{+,\mathbf{r}} \hat{p}^\dagger_{-,\mathbf{r}} \hat{p}_{-,\mathbf{r}} \hat{p}_{+,\mathbf{r}} \right). \tag{S8}$$

Here, $U_s = U_0 \int d\mathbf{r} |w_s(\mathbf{r})|^4$ and $U_p = U_0 \int d\mathbf{r} |w_+(\mathbf{r})|^4$. The interaction parameter U_0 is positive for repulsive collisions. We note that some terms, such as $\hat{p}^\dagger_{+,\mathbf{r}} \hat{p}^\dagger_{+,\mathbf{r}} \hat{p}_{-,\mathbf{r}} \hat{p}_{-,\mathbf{r}}$ and $\hat{p}^\dagger_{+,\mathbf{r}} \hat{p}^\dagger_{+,\mathbf{r}} \hat{p}_{+,\mathbf{r}} \hat{p}_{-,\mathbf{r}}$ do not occur because of the orbital angular momentum conservation during two-body collisions. The interaction Hamiltonian can also be rewritten as

$$\hat{H}_{\text{int}} = \frac{U_s}{2} \sum_{\mathbf{r}\in B} \hat{n}_{s,\mathbf{r}} \left(\hat{n}_{s,\mathbf{r}} - 1 \right) + \frac{3U_p}{4} \sum_{\mathbf{r}\in A} \left[\hat{n}_{p,\mathbf{r}} \left(\hat{n}_{p,\mathbf{r}} - \frac{2}{3} \right) - \frac{1}{3} \hat{L}^2_{z,\mathbf{r}} \right]. \tag{S9}$$

Here, $\hat{n}_{s,\mathbf{r}} = \hat{s}^\dagger_{\mathbf{r}} \hat{s}_{\mathbf{r}}$, $\hat{n}_{p,\mathbf{r}} = \hat{p}^\dagger_{+,\mathbf{r}} \hat{p}_{+,\mathbf{r}} + \hat{p}^\dagger_{-,\mathbf{r}} \hat{p}_{-,\mathbf{r}}$, and $\hat{L}_{z,\mathbf{r}} = \hat{p}^\dagger_{+,\mathbf{r}} \hat{p}_{+,\mathbf{r}} - \hat{p}^\dagger_{-,\mathbf{r}} \hat{p}_{-,\mathbf{r}}$ represents the orbital angular momentum.

S-4 Chiral condensate

Experimentally, we load atoms into the second band of the BN lattice and investigate the subsequent condensation dynamics. We first consider the single-particle part of the Hamiltonian. The second band minima are located at the K_Δ and K_∇ points. Using the tight-binding model description, the corresponding Bloch functions are

$$\begin{aligned} \Phi_{K_\Delta}(\mathbf{r}) &= e^{i\mathbf{K}_\Delta \cdot \mathbf{r}} \left(\cos\xi_0, \sin\xi_0, 0 \right)^T, \\ \Phi_{K_\nabla}(\mathbf{r}) &= e^{i\mathbf{K}_\nabla \cdot \mathbf{r}} \left(\cos\xi_0, 0, \sin\xi_0 \right)^T. \end{aligned} \tag{S10}$$

Here, we choose $\mathbf{K}_\Delta = k_L(0,1)$ and $\mathbf{K}_\nabla = k_L(0,-1)$. $\xi_0 = \arctan[6t/(\sqrt{\delta^2 + 36t^2} - \delta)]$ with $\delta = \varepsilon_s - \varepsilon_p$. This double-well degeneracy is protected by the time-reversal symmetry of the system and $\Phi_{K_\Delta}(\mathbf{r}) = \mathcal{T} \Phi_{K_\nabla}(\mathbf{r})$, where $\mathcal{T}$ is the time-reversal operator.

Including the interaction between the atoms, we next search for the mean-field ground state for the Bose-Hubbard model to describe the condensate formation after loading atoms into

the second band. For weak interactions, it is reasonable to maintain a description in terms of condensates at the two quasi-momenta associated with the K_Δ and K_∇ points. Therefore, we assume that the condensate order parameter $\langle\hat{\Psi}(\mathbf{r})\rangle \equiv (\langle\hat{s}_\mathbf{r}\rangle, \langle\hat{p}_{+,\mathbf{r}}\rangle, \langle\hat{p}_{-,\mathbf{r}}\rangle)^T$ can be written as

$$\langle\hat{\Psi}(\mathbf{r})\rangle = \begin{pmatrix} \sqrt{\rho_{s,K_\Delta}}e^{i\theta_{s,K_\Delta}}e^{i\mathbf{K}_\Delta\cdot\mathbf{r}} + \sqrt{\rho_{s,K_\nabla}}e^{i\theta_{s,K_\nabla}}e^{i\mathbf{K}_\nabla\cdot\mathbf{r}} \\ \sqrt{\rho_{p_+,K_\Delta}}e^{i\theta_{p_+,K_\Delta}}e^{i\mathbf{K}_\Delta\cdot\mathbf{r}} + \sqrt{\rho_{p_+,K_\nabla}}e^{i\theta_{p_+,K_\nabla}}e^{i\mathbf{K}_\nabla\cdot\mathbf{r}} \\ \sqrt{\rho_{p_-,K_\Delta}}e^{i\theta_{p_-,K_\Delta}}e^{i\mathbf{K}_\Delta\cdot\mathbf{r}} + \sqrt{\rho_{p_-,K_\nabla}}e^{i\theta_{p_-,K_\nabla}}e^{i\mathbf{K}_\nabla\cdot\mathbf{r}} \end{pmatrix}. \tag{S11}$$

Here, we suspend the restriction that the atoms can only condense in the second band, because interaction may induce higher band populations.

The mean-field energy for the interaction Hamiltonian is given by $E^{\text{int}}_{\text{MF}} = E^s_{\text{MF}} + E^p_{\text{MF}}$, where E^s_{MF} and E^p_{MF} describe the s-orbital and p-orbital interaction, respectively, and are given by

$$E^s_{\text{MF}} = \frac{U_s}{2}(\rho^2_{s,K_\Delta} + 4\rho_{s,K_\Delta}\rho_{s,K_\nabla} + \rho^2_{s,K_\nabla}), \tag{S12}$$

$$\begin{aligned} E^p_{\text{MF}} = {} & \frac{U_p}{2}(\rho^2_{p_+,K_\Delta} + 4\rho_{p_+,K_\Delta}\rho_{p_-,K_\Delta} + \rho^2_{p_-,K_\Delta}) + \frac{U_p}{2}(\rho^2_{p_+,K_\nabla} + 4\rho_{p_+,K_\nabla}\rho_{p_-,K_\nabla} + \rho^2_{p_-,K_\nabla}) \\ & +2U_p\left(\rho_{p_+,K_\Delta}\rho_{p_+,K_\nabla} + \rho_{p_-,K_\Delta}\rho_{p_-,K_\Delta} + \rho_{p_+,K_\Delta}\rho_{p_-,K_\nabla} + \rho_{p_-,K_\Delta}\rho_{p_+,K_\nabla}\right) \\ & +4U_p\text{Re}\left[\sqrt{\rho_{p_+,K_\Delta}\rho_{p_-,K_\nabla}\rho_{p_+,K_\nabla}\rho_{p_-,K_\Delta}}e^{i\left(-\theta_{p_+,K_\Delta}-\theta_{p_-,K_\nabla}+\theta_{p_+,K_\nabla}+\theta_{p_-,K_\Delta}\right)}\right]. \end{aligned} \tag{S13}$$

Minimizing the total energy $E_{\text{MF}} = E^0_{\text{MF}} + E^s_{\text{MF}} + E^p_{\text{MF}}$ of the system, where E^0_{MF} is the mean-field energy for the single-particle part, under a fixed total atom number N, we can determine the unknown parameters in the ansatz. Here, the atom number is given by

$$N = N_u \sum_{\alpha=s,p_+,p_-}\sum_{\beta=K_\Delta,K_\nabla}\rho_{\alpha,\beta}, \tag{S14}$$

where N_u is the number of unit cells. We find that the final ground state takes the same form as Eq. (S10) and can be written as

$$\langle\hat{\Psi}(\mathbf{r})\rangle = \sqrt{\rho}e^{i\mathbf{K}_\Delta\cdot\mathbf{r}}\left(\cos\xi, \sin\xi, 0\right)^{\text{T}} \tag{S15}$$

or

$$\langle\hat{\Psi}(\mathbf{r})\rangle = \sqrt{\rho}e^{i\mathbf{K}_\nabla\cdot\mathbf{r}}\left(\cos\xi, 0, \sin\xi\right)^{\text{T}}. \tag{S16}$$

We denote these two degenerate state as $|K_\Delta\rangle$ and $|K_\nabla\rangle$, respectively. Here, the parameter ξ is determined by the numerical optimization and is usually different from the single-particle case $\xi \neq \xi_0$. This means that the interaction induces higher-band populations in the ground state. For very week interaction $\hat{H}_{\text{int}} \to 0$, the ground state order parameter is proportional to the Bloch wave function at a single minimum of the second band of the BN lattice with $\xi \to \xi_0$. In summary, the interaction among atoms induces a spontaneous symmetry-breaking leading to a condensate at a single band minimum.

In order to numerically determine the Bloch functions at the minima of the second band, a plane-wave expansion is applied. Considering the case when atoms condense at the K_Δ point of the second band, the averaged angular momentum per atom is given by

$$\langle \hat{L}_z \rangle / N = \frac{\int \Phi^*_{K_\Delta}(x,y) \hat{L}_z \Phi_{K_\Delta}(x,y) dxdy}{\int |\Phi_{K_\Delta}(x,y)|^2 dxdy}, \tag{S17}$$

where $\hat{L}_z = -i\hbar(x\partial_y - y\partial_x)$ denotes the angular momentum operator. Figure S4 shows the numerically calculated expectation value of the angular momentum per atom for fixed V_2 and varying V_1. For the case with $(V_1, V_2) = (7.81, 7.23)\, E_{\text{R}}$, most of atoms in the second band reside in local s-orbitals in the shallow wells, which leads to a tiny angular momentum for the chiral condensate. In contrast, as we lower the energy offset of the p-orbitals by raising V_1 up to $8.35\, E_{\text{R}}$, a significant amount of atoms are transferred to these p-orbitals, leading to a pronounced increase of angular momentum.

S-5 $\mathcal{T}$-invariant condensate

By minimizing the mean-field energy E_{MF} with a fixed particle number N, we find that the atoms should condense at either the K_Δ or the K_∇ point of the first BZ. Each state no longer respects the $\mathcal{T}$ and D_3 point-group symmetry. One may thus expect that after loading atoms into the second band of the BN lattice, they should directly condense at a single K point.

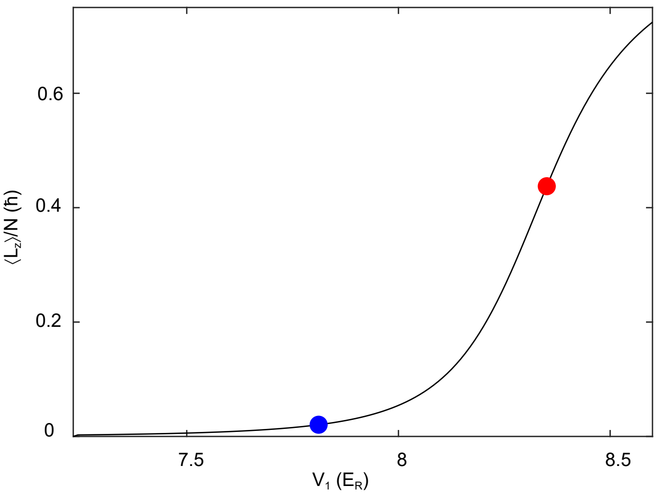

Figure S4: The averaged angular momentum per atom for the condensate at K_Δ of the second band with varying V_1. Here, $V_2 = 7.23 E_\mathrm{R}$. Blue (Red) dot refers to $V_1 = 7.81\, E_\mathrm{R}$ ($8.35\, E_\mathrm{R}$).

The experiment, however, shows that the formed condensate in general has two momentum components K_Δ and K_∇ with a fluctuating population ratio, even though this is not the lowest-energy state. Here, we put forward an explanation that it is owing to symmetry constraints of the system, which make it difficult for bosons to condense at just a single K point.

In our experiment, a Bose-Einstein condensate (BEC) of ^{87}Rb atoms is first prepared in the Γ-point of the BN optical lattice. In a rapid quench using the population swapping technique, the atoms then are excited to the Γ-point of the second band. Subsequently, atoms start to re-condense in the second band. During this process, the quasi-momentum of the system should be conserved. Since the initial state has zero total quasi-momentum, we first assume that the atoms condense at momenta $\mathbf{k}$ and $-\mathbf{k}$ with equal populations to satisfy the quasi-momentum conservation. Here, $\mathbf{k}$ is assumed not to coincide with the inversion invariant points Γ and M. Therefore, the corresponding order parameter is given by

$$\langle \hat{\Psi}(\mathbf{r}) \rangle = \begin{pmatrix} \sqrt{\rho_{s,k}} e^{i\theta_{s,k}} e^{i\mathbf{k}\cdot\mathbf{r}} + \sqrt{\rho_{s,-k}} e^{i\theta_{s,-k}} e^{-i\mathbf{k}\cdot\mathbf{r}} \\ \sqrt{\rho_{p_+,k}} e^{i\theta_{p_+,k}} e^{i\mathbf{k}\cdot\mathbf{r}} + \sqrt{\rho_{p_+,-k}} e^{i\theta_{p_+,-k}} e^{-i\mathbf{k}\cdot\mathbf{r}} \\ \sqrt{\rho_{p_-,k}} e^{i\theta_{p_-,k}} e^{i\mathbf{k}\cdot\mathbf{r}} + \sqrt{\rho_{p_-,-k}} e^{i\theta_{p_-,-k}} e^{-i\mathbf{k}\cdot\mathbf{r}} \end{pmatrix}. \tag{S18}$$

Minimizing the total mean-field energy, we find that $\mathbf{k}$ should be equal to $\mathbf{K}_\Delta$ or $\mathbf{K}_\nabla$ for the ground state with an order parameter

$$\langle\hat{\Psi}(\mathbf{r})\rangle = e^{i\theta'}\sqrt{\rho_K}e^{i\mathbf{K}_\Delta\cdot\mathbf{r}}\begin{pmatrix}\cos\xi'\\ \sin\xi'\\ 0\end{pmatrix} + e^{-i\theta'}\sqrt{\rho_K}e^{i\mathbf{K}_\nabla\cdot\mathbf{r}}\begin{pmatrix}\cos\xi'\\ 0\\ \sin\xi'\end{pmatrix}. \tag{S19}$$

This state is an equal superposition of two momentum states at the K_Δ and K_∇ points and is invariant under time-reversal. We denote this state as $|K_\Delta, K_\nabla\rangle$.

Next, we consider the case where the atoms condense at the inversion-invariant points Γ and the three inequivalent M points. In contrast to the discussion above, the atoms can condense at a single or several inversion-invariant points, because of the existence of the two-particle interaction term $\hat{\Psi}_M^\dagger\hat{\Psi}_M^\dagger\hat{\Psi}_\Gamma\hat{\Psi}_\Gamma$. Since the energy for the Γ point of the second band is much larger than the energy for the three M points, we consider only the possibility of condensation at the M points. Assuming that the atoms condense at one of the three degenerate M points, leading to a $\mathcal{T}$-invariant state, the corresponding order parameter for a specific choice of one of the degenerate states, denoted as $|M\rangle$, takes the form

$$\langle\hat{\Psi}(\mathbf{r})\rangle = \sqrt{\rho_M}e^{i\mathbf{M}\cdot\mathbf{r}}\begin{pmatrix}e^{i\frac{\pi}{3}}\cos\xi''\\ (-\sin\xi'')/\sqrt{2}\\ (-\sin\xi'')/\sqrt{2}\end{pmatrix}, \tag{S20}$$

where ξ'' varies with the system parameters.

We further compare the mean-field energy for different order parameters by considering the lattice potential used in the experiment with $(V_1, V_2) = (7.81, 7.23)\,E_\mathrm{R}$. Figure S5 shows the numerical results. Although $|K_\Delta\rangle$ or $|K_\nabla\rangle$ is always the lowest energy state, we find that due to the quasi-momentum conservation, the $|K_\Delta, K_\nabla\rangle$ state is the true ground state for the weakly interacting case. This is consistent with our experimental observation that during the initial stage of the time evolution, the atoms prefer to condense at K_Δ and K_∇ points with roughly equal populations. If, however, the interaction is larger than a critical value, the $|M\rangle$ state has a lower energy. Furthermore, we numerically find that a specific condensation at two out of

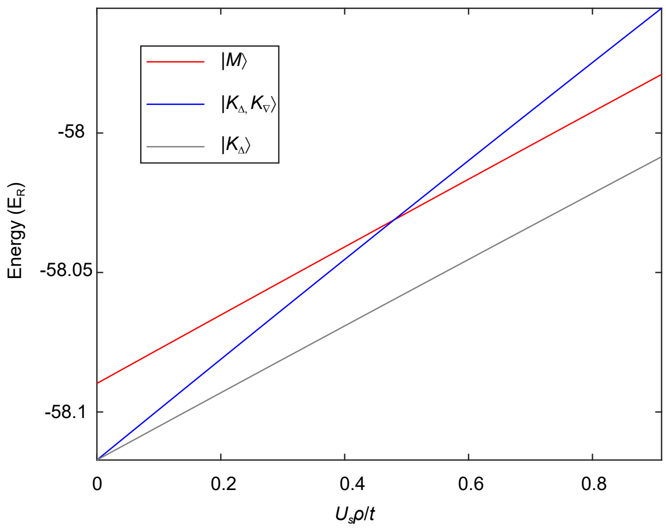

Figure S5: Calculated mean-field energy for the $|M\rangle$ state (red line), $|K_\Delta, K_\nabla\rangle$ state (blue line) and $|K_\Delta\rangle$ state (gray line) as a function of $U_s\rho/t$, which characterize the strength of interaction.

three M points has an even lower energy with a tiny difference. This state is similar to the $\mathcal{T}$-symmetry breaking ground state found in the p-band of the triangular lattice [2]. Nevertheless, our experimental results indicate that the interaction in our system is relatively weak, which facilitates the observation of chiral condensates.

S-6 Topological Bogoliubov excitations

In condensed matter physics, the evidence for Majorana fermions has been mostly obtained from transport measurements, which rely heavily on Bogoliubov-de Gennes (BdG) equations (for superconductivity) as the established theoretical model for quasiparticle excitations. The other important signature of topological superconductors, i.e., a time-reversal symmetry breaking complex order parameter of Cooper pairing, is often difficult or even impossible to probe in electronic materials. In our case of cold gas physics, momentum spectroscopy, in combination with the particular lattice geometry, enables us to unambiguously measure the order parameter and to observe the time reversal symmetry breaking signature in the course of its formation.

However, transport measurements are not easily possible here to confirm a non-trivial topology. For concluding on the topological nature of the state formed in the present work, we employ the standard Bogoliubov analysis. This theory is well established and elementary and leaves no doubt about the resulting conclusions.

The quasi-momentum conservation impedes the formation of a time-reversal symmetry broken condensate in the initial stage of atom re-condensation in the second band. However, as we observed experimentally, in the long-time limit of the re-condensation process, due to evaporative cooling and associated loss of atoms along the direction of gravity, angular momentum can be exchanged with the environment and a time-reversal symmetry broken condensate arises. Here, we investigate the Bogoliubov excitations on top of the chiral condensate and find that there are topologically nontrivial excitations. We select one degenerate ground state with bosons condensed at the K_Δ point. The corresponding state exhibits positive orbital angular momenta. The analogous K_∇ condensate with negative orbital angular momentum has similar excitation spectra and topological properties. Applying the usual Bogoliubov method, we obtain the BdG Hamiltonian that characterizes the Bogoliubov excitations on top of the state $|K_\Delta\rangle$, which reads

$$\hat{H}_{\mathrm{BdG}} = \frac{1}{2} \sum_{\mathbf{k} \neq \mathbf{K}_\Delta} \left(\hat{\Psi}_{\mathbf{k}}^\dagger, \hat{\Psi}_{2\mathbf{K}_\Delta - \mathbf{k}}^{\mathrm{T}} \right) \mathcal{H}_{\mathrm{BdG}}(\mathbf{k}) \begin{pmatrix} \hat{\Psi}_{\mathbf{k}} \\ \hat{\Psi}_{2\mathbf{K}_\Delta - \mathbf{k}}^{\dagger \mathrm{T}} \end{pmatrix}. \tag{S21}$$

Here, the field operator is given by $\hat{\Psi}_{\mathbf{k}} = (\hat{s}_{\mathbf{k}}, \hat{p}_{+,\mathbf{k}}, \hat{p}_{-,\mathbf{k}})^T$ and the Hermitian matrix $\mathcal{H}_{\mathrm{BdG}}(\mathbf{k})$ takes the form of

$$\mathcal{H}_{\mathrm{BdG}}(\mathbf{k}) = \begin{pmatrix} h_a(\mathbf{k}) & h_x(\mathbf{k}) \\ h_x^*(2\mathbf{K}_\Delta - \mathbf{k}) & h_b(\mathbf{k}) \end{pmatrix} \tag{S22}$$

with $h_b(\mathbf{k}) = h_a^*(2\mathbf{K}_\Delta - \mathbf{k})$. $\mathcal{H}_{\mathrm{BdG}}(\mathbf{k})$ has a particle-hole symmetry $\tau_x \mathcal{H}_{\mathrm{BdG}}^*(2\mathbf{K}_\Delta - \mathbf{k}) \tau_x = \mathcal{H}_{\mathrm{BdG}}(\mathbf{k})$ with $\tau_x = \sigma_x \otimes \mathbb{1}_{3\times3}$, where σ_x is Pauli matrix and $\mathbb{1}_{3\times3}$ is the identity matrix. The concrete expression of the matrix $h_a(\mathbf{k})$ is given by

$$h_a(\mathbf{k}) = \mathcal{H}_0(\mathbf{k}) - \mu + \begin{pmatrix} 2U_s\rho\cos^2\xi & & \\ & 2U_p\rho\sin^2\xi & \\ & & 2U_p\rho\sin^2\xi \end{pmatrix}. \tag{S23}$$

Here, $\mu = U_s \rho \cos^4 \xi + U_p \rho \sin^4 \xi + E^0_{\mathbf{K}_\Delta}$ with $E^0_{\mathbf{K}_\Delta}$ being the single-particle energy of the ground state and the density ρ being the atom number per unit cell. $\mathcal{H}_0(\mathbf{k})$ and $h_a(\mathbf{k})$ have the same degenerate quadratic band crossing point and their degenerate energies are ε_p and $\varepsilon_p - \mu + 2U_p\rho \sin^2 \xi$, respectively. The matrix $h_x(\mathbf{k})$ is given by

$$h_x(\mathbf{k}) = \begin{pmatrix} U_s\rho\cos^2\xi & & \\ & U_p\rho\sin^2\xi & \\ & & 0 \end{pmatrix}, \tag{S24}$$

which is purely originated from atom interactions.

The Bogoliubov excitations can be solved by a Bogoliubov transformation $T^\dagger_{\mathbf{k}} \mathcal{H}_{\mathrm{BdG}}(\mathbf{k}) T_{\mathbf{k}} = E_{\mathbf{k}}$, where $E_{\mathbf{k}}$ is a diagonal matrix and pseudo-unitary matrix $T_{\mathbf{k}}$ satisfies $T^\dagger_{\mathbf{k}} \tau_z T_{\mathbf{k}} = \tau_z$ with $\tau_z = \sigma_z \otimes \mathbb{1}_{3\times 3}$. The excitation spectra are included in the diagonal elements of $E_{\mathbf{k}}$, which are plotted in Fig. S6. We note that a gap close to the quadratic band crossing point of the single-particle spectra is opened in the excitation spectra. This is due to the time-reversal symmetry breaking of the $|K_\Delta\rangle$ state.

With the gap opened, we are able to study the topological features for each individual excitation band. Following our previous works on characterizing topological bosonic Bogoliubov excitations [3, 4], we introduce an effective non-interacting Hamiltonian $\mathcal{H}_{\text{eff}}(\mathbf{k})$ that shares the identical symmetry, spectra and topological properties as the original BdG Hamiltonian $\mathcal{H}_{\mathrm{BdG}}(\mathbf{k})$. Specifically, the effective block-diagonal Hamiltonian is obtained by a contractible Bogoliubov transformation,

$$\mathcal{H}_{\text{eff}}(\mathbf{k}) = e^W \mathcal{H}_{\mathrm{BdG}}(\mathbf{k}) e^W \tag{S25}$$

with

$$e^{2W} = \mathcal{H}_{\mathrm{BdG}}^{-\frac{1}{2}} (\mathcal{H}_{\mathrm{BdG}}^{\frac{1}{2}} \tau_z \mathcal{H}_{\mathrm{BdG}} \tau_z \mathcal{H}_{\mathrm{BdG}}^{\frac{1}{2}})^{\frac{1}{2}} \mathcal{H}_{\mathrm{BdG}}^{-\frac{1}{2}}. \tag{S26}$$

Here, $\mathcal{H}_{\text{eff}}(\mathbf{k}) = h_0(\mathbf{k}) \oplus h'_0(\mathbf{k})$, and has the particle-hole symmetry such that $h'_0(\mathbf{k}) = h^*_0(2\mathbf{K}_1 - \mathbf{k})$. Therefore, the topology of the BdG Hamiltonian $\mathcal{H}_{\mathrm{BdG}}(\mathbf{k})$ is completely equivalent to the number-conserved non-interacting system $h_0(\mathbf{k})$.

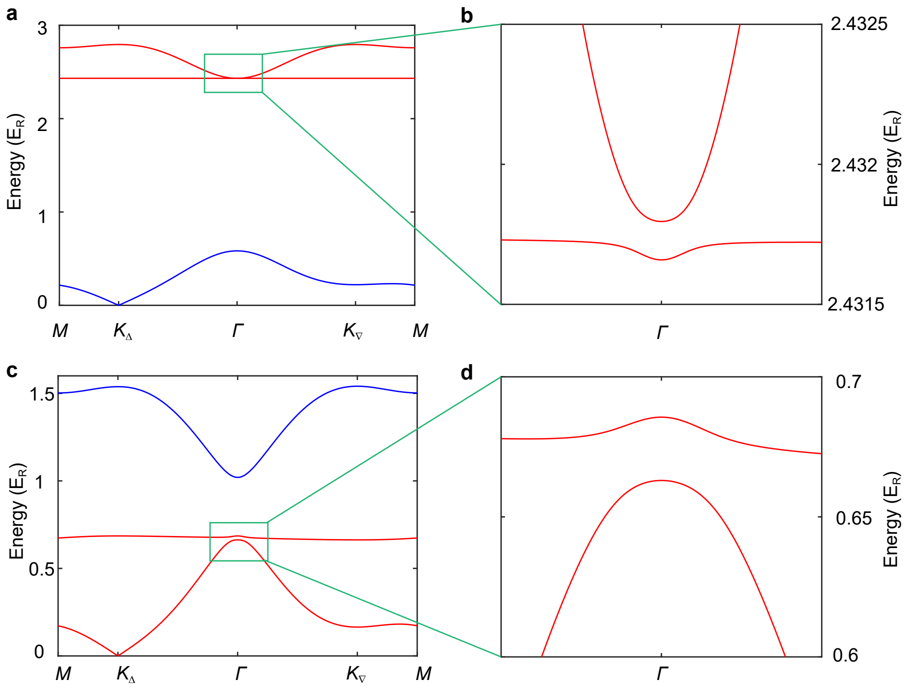

Figure S6: (**a**) The excitation spectrum in the case of $\delta < 0$. The numerical parameters are chosen as $\varepsilon_s = -57.8488E_\mathrm{R}$, $\varepsilon_p = -55.3149E_\mathrm{R}$, $t = 0.2891E_\mathrm{R}$, $U_s\rho = 0.5270E_R$, and $U_p\rho = 0.2246E_R$. (**c**) The excitation spectrum in the case of $\delta > 0$. The numerical parameters are chosen as $\varepsilon_s = -58.3315E_R$, $\varepsilon_p = -58.6837E_R$, $t = 0.2213E_R$, $U_s\rho = 0.4682E_R$, and $U_p\rho = 0.2780E_R$. (**b**, **d**) Expanded view on the green boxes in (**a**, **c**).

The topological feature of $h_0(\mathbf{k})$ can be characterized by the standard Chern number. Based on the unitary diagonalization $U_\mathbf{k}^\dagger h_0(\mathbf{k})U_\mathbf{k} = D_\mathbf{k}$ by the diagonal matrix $D_\mathbf{k}$ and the unitary matrix $U_\mathbf{k}$, the Berry connection for the j-th band is defined by

$$A_{j,\nu}(\mathbf{k}) = -i\langle U_\mathbf{k}^j|\partial_{k_\nu}|U_\mathbf{k}^j\rangle,\ (\nu = x, y), \tag{S27}$$

where $U_\mathbf{k}^j$ is the j-th column of $U_\mathbf{k}$. Then the Chern number of the j-th band is given by

$$\mathcal{C}_j = \frac{1}{2\pi}\int d\mathbf{k}B_j(\mathbf{k}), \tag{S28}$$

where the Berry curvature reads

$$B_j(\mathbf{k}) = \partial_{k_x}A_{j,y}(\mathbf{k}) - \partial_{k_y}A_{j,x}(\mathbf{k}). \tag{S29}$$

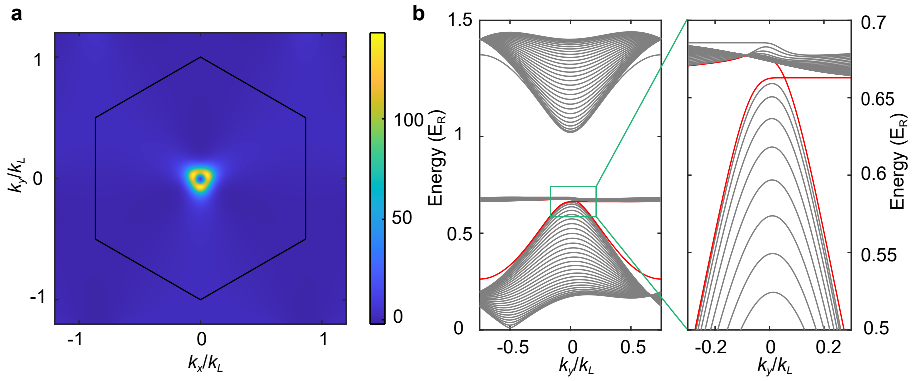

Figure S7: (**a**) Berry curvature for the lowest excitation band. The black solid honeycomb indicates the first BZ. (**b**) Bogoliubov excitation spectra for the finite system with the expanded view on the right. The red solid lines indicate the edge-state dispersion. The parameters are chosen as in Fig. S6 (**c**).

We calculate the Berry curvatures and the Chern numbers for all bands numerically. The Berry curvature of the lowest band is shown in Fig. S7 (**a**) with the associated Chern number $\mathcal{C}_1 = 1$. This nonzero topological invariant arises from the gap opening close to the quadratic band crossing point. Furthermore, we also calculate the Bogoliubov excitation spectra for a finite-lattice system. A periodic boundary condition is applied along the y direction and 30 unit cells are taken along the x direction. We observe topological edge excitations as shown in Fig. S7 (**b**), which are consistent with the common knowledge of the bulk-boundary correspondence.

Although we have transformed the original BdG Hamiltonian $\mathcal{H}_{\mathrm{BdG}}(\mathbf{k})$ into the effective non-interacting Hamiltonian $h_0(\mathbf{k})$ precisely, this transformation is too complex to understand the emergence of the topological feature at first sight. Considering that the topological properties of our system is principally determined by the quadratic band crossing point and the interaction is week enough, we can only focus on the nearby region of the quadratic band crossing point and treat $h_x(\mathbf{k})$ as a perturbation. Then an approximate formula of $h_0(\mathbf{k})$ can be derived

by applying a powerful perturbation method discussed in Ref. [4] as

$$h_0(\mathbf{k}) \approx h_a(\mathbf{k}) - \left[h_x(\lambda \mathbb{1}_{3\times 3} + h_b)^{-1} h_x^\dagger\right]_{\mathbf{k}=\mathbf{p}}, \tag{S30}$$

where $\lambda = \varepsilon_p - \mu + 2U_p\rho \sin^2 \xi$ and $\mathbf{p} = (0,0)$ are the energy and the quasi-momentum of the degenerate point for $h_a(\mathbf{k})$ respectively. After substituting $\mathbf{p}$ and λ into the formula, we can obtain the effective model of $h_0(\mathbf{k})$ as

$$h_0(\mathbf{k}) \approx h_a(\mathbf{k}) - \begin{pmatrix} \frac{2u_2 v_1^2}{-9t^2+2u_2(u_1+u_2)} & & \\ & \frac{v_2^2}{2u_2} & \\ & & 0 \end{pmatrix}, \tag{S31}$$

where $v_1 = U_s\rho\cos^2\xi$, $v_2 = U_p\rho\sin^2\xi$, $u_1 = \varepsilon_s - \mu + 2v_1$, and $u_2 = \varepsilon_p - \mu + 2v_2$. Obviously, the last term lifts the degeneracy of p orbitals with $v_2^2/(2u_2) \neq 0$. It indicates that the matrix $h_x(\mathbf{k})$ originating from interaction opens the gap at the quadratic band crossing point for the Bogoliubov excitations.

We further try to reduce $h_0(\mathbf{k})$ to an effective two-band model to understand the topological properties of the gap opened at the quadratic band crossing point explicitly. Since the quadratic band crossing point in the single-particle energy spectra mainly originates from two p orbitals, we focus on this subspace and treat the influence of the s orbital as a perturbation. By integrating out the degree of freedom of s orbital, we obtain an effective two-band Hamiltonian $\tilde{h}_0(\mathbf{k})$ with the form of $\tilde{h}_0(\mathbf{k}) = \mathbf{d}(\mathbf{k}) \cdot \boldsymbol{\sigma}$, where the vectors $\mathbf{d}(\mathbf{k})$ and $\boldsymbol{\sigma}$ are defined as $\mathbf{d}(\mathbf{k}) = (d_0(\mathbf{k}), d_1(\mathbf{k}), d_2(\mathbf{k}), d_3(\mathbf{k}))$ and $\boldsymbol{\sigma} = (\sigma_0, \sigma_1, \sigma_2, \sigma_3)$ respectively. For the non-interacting system, the vector $\mathbf{d}(\mathbf{k})$ is give by $d_0(\mathbf{k}) = t_0(k_x^2 + k_y^2)$, $d_1(\mathbf{k}) = t_1(k_x^2 - k_y^2)$, $d_2(\mathbf{k}) = 2t_2 k_x k_y$, and $d_3(\mathbf{k}) = 0$, where $t_0 = t_1 = t_2 = 9t^2 a_0^2/[4(\varepsilon_p - \varepsilon_s)]$. Therefore, the energy spectra are gapless for lacking the σ_3 term in $\tilde{h}_0(\mathbf{k})$. In contrast, when we consider the interacting system we find that $h_x(\mathbf{k})$ induces a new σ_3 term in the effective Hamiltonian $\tilde{h}_0(\mathbf{k})$ with $d_3(\mathbf{k}) = -v_2^2/(4u_2)$ (see Eq. S31), which breaks the time-reversal symmetry giving rise to topological nontrivial Bogoliubov excitations.

S-7 Relation between this work and previous work in a chequerboard lattice

The occurrence of a topological excitation spectrum is intimately connected with the circumstance that, in the hexagonal BN-lattice studied here, there is a true spontaneous time-reversal symmetry breaking into a globally chiral state, in contrast to the work in a chequerboard lattice in Ref. [5]. In the earlier work, the observed order was microscopically (on a spatial scale of a unit cell of the lattice) violating time-reversal symmetry. However, the combination of the operation of time-reversal with a translation by a primitive lattice vector has left the system invariant. This subtle difference in symmetry leads to qualitatively different quantum states – with globally chiral, ferromagnetic order in this work and locally chiral, antiferromagnetic order in Ref. [5].

There is a second subtle but remarkable difference between this work and Ref. [5]: As discussed in the main text, in this work, an unambiguous signature of global time-reversal symmetry breaking and globally chiral order can be directly extracted from a simple momentum spectrum. Essentially possible phase separation scenarios etc. can be unequivocally ruled out. This is not the case in Ref. [5], where additional phase information is required to identify the order parameter, which cannot be derived from a momentum spectrum. The difference arises from the fundamental dissimilarity of the interplay between symmetry of the lattice potentials and degeneracy of p-orbitals in the second band. The chequerboard lattice potential combines a fourfold discrete rotation symmetry with two degenerate p-orbitals in the second band. In contrast, the BN-lattice potential provides a threefold discrete rotation symmetry, which leads to a more intricate interplay with the twofold degeneracy of the p-orbitals in the second band. As a result, the emerging globally chiral state in the present work is associated with a condensate located at a single high symmetry point in quasi-momentum space (one of the two K points, see

Fig. 1b of the main text) and thus possesses a unique momentum spectrum. In contrast, the locally chiral state, formed in the work in Ref. [5], is a superposition of two condensate fractions with a relative phase of $\pi/2$, located at different X-points of the chequerboard lattice. Only in a later interference experiment in Ref. [6], it could be shown that in fact a fixed relative phase prevailed in Ref. [5]. Its value $\pi/2$ could only be determined from additional basic theoretical analysis Ref. [7].

References

[1] Marzari, N. & Vanderbilt, D. Maximally localized generalized wannier functions for composite energy bands. *Phys. Rev. B* **56**, 12847–12865 (1997).

[2] Wu, C., Liu, W.-V., Moore, J. & Das Sarma, S. Quantum stripe ordering in optical lattices. *Phys. Rev. Lett.* **97**, 190406 (2006).

[3] Zhou, Z., Wan, L.-L. & Xu, Z.-F. Topological classification of excitations in quadratic bosonic systems. *Journal of Physics A: Mathematical and Theoretical* **53**, 425203 (2020).

[4] Wan, L.-L., Zhou, Z. & Xu, Z.-F. Squeezing-induced topological gap opening on bosonic bogoliubov excitations. *Phys. Rev. A* **103**, 013308 (2021).

[5] Wirth, G., Ölschläger, M. & Hemmerich, A. Evidence for orbital superfluidity in the p-band of a bipartite optical. *Nat. Phys.* **73**, 147–153 (2011).

[6] Kock, T. *et al.* Observing chiral superfluid order by matter-wave interference. *Phys. Rev. Lett.* **114**, 115301 (2015).

[7] Kock, T., Hippler, C., Ewerbeck, A. & Hemmerich, A. Orbital optical lattices with bosons. *Journal of Physics B: Atomic, Molecular and Optical Physics* **49**, 042001 (2016).
